# *Papaver* Plants: Current Insights on Phytochemical and Nutritional Composition Along with Biotechnological Applications

**DOI:** 10.1155/2022/2041769

**Published:** 2022-02-03

**Authors:** Monica Butnariu, Cristina Quispe, Jesús Herrera-Bravo, Marius Pentea, Ioan Sarac, Aylin Seylam Küşümler, Beraat Özçelik, Sakshi Painuli, Prabhakar Semwal, Muhammad Imran, Tanweer Aslam Gondal, Simin Emamzadeh-Yazdi, Natallia Lapava, Zubaida Yousaf, Manoj Kumar, Ali Hussein Eid, Yusra Al-Dhaheri, Hafiz Ansar Rasul Suleria, María del Mar Contreras, Javad Sharifi-Rad, William C. Cho

**Affiliations:** ^1^Banat's University of Agricultural Sciences and Veterinary Medicine “King Michael I of Romania” from Timisoara, Timisoara, Romania; ^2^Facultad de Ciencias de la Salud, Universidad Arturo Prat, Avda. Arturo Prat 2120, Iquique 1110939, Chile; ^3^Departamento de Ciencias Básicas, Facultad de Ciencias, Universidad Santo Tomas, Chile; ^4^Center of Molecular Biology and Pharmacogenetics, Scientific and Technological Bioresource Nucleus, Universidad de La Frontera, Temuco 4811230, Chile; ^5^İstanbul Okan University, Nutrition and Dietetics Department, Tuzla, İstanbul, Turkey; ^6^Department Food Engineering, Faculty of Chemical and Metallurgical Engineering, Istanbul Technical University, Maslak, 34469 Istanbul, Turkey; ^7^BIOACTIVE Research & Innovation Food Manufacturing Industry Trade LTD Co., Maslak, Istanbul 34469, Turkey; ^8^Department of Biotechnology, Graphic Era University, 248001, Dehradun, Uttarakhand, India; ^9^Himalayan Environmental Studies and Conservation Organization, Prem Nagar, Dehradun, 248001 Uttarakhand, India; ^10^Department of Life Sciences, Graphic Era Deemed to be University, Dehradun-248002, Uttarakhand, India; ^11^University Institute of Diet and Nutritional Sciences, Faculty of Allied Health Sciences, The University of Lahore-Lahore, Pakistan; ^12^School of Exercise and Nutrition, Deakin University, Victoria 3125, Australia; ^13^Department of Plant and Soil Sciences, University of Pretoria, Gauteng 0002, South Africa; ^14^Medicine Standardization Department of Vitebsk State Medical University, Belarus; ^15^Department of Botany, Jail Road, Lahore 54000, Pakistan; ^16^Chemical and Biochemical Processing Division, ICAR-Central Institute for Research on Cotton Technology, 400019, Mumbai, India; ^17^Department of Basic Medical Sciences, College of Medicine, QU Health, Qatar University, PO Box 2713, Doha, Qatar; ^18^Biomedical and Pharmaceutical Research Unit, QU Health, Qatar University, PO Box 2713, Doha, Qatar; ^19^Department of Biology, College of Science, United Arab Emirates University, UAE; ^20^Department of Agriculture and Food Systems, The University of Melbourne, Melbourne 3010, Australia; ^21^Department of Chemical, Environmental and Materials Engineering, Universidad de Jaén, Campus las Lagunillas, s/n, 23071 Jaén, Spain; ^22^Facultad de Medicina, Universidad del Azuay, Cuenca, Ecuador; ^23^Department of Clinical Oncology, Queen Elizabeth Hospital, Kowloon, Hong Kong

## Abstract

The genus *Papaver* is highly esteemed in the pharmacy industry, in the culinary field, and as ornamental plants. These plants are also valued in traditional medicine. Among all *Papaver* species, *Papaver somniferum* L. (opium poppy) is the most important species in supplying phytochemicals for the formulation of drugs, mainly alkaloids like morphine, codeine, rhoeadine, thebaine, and papaverine. In addition, *Papaver* plants present other types of phytochemicals, which altogether are responsible for its biological activities. Therefore, this review covers the phytochemical composition of *Papave*r plants, including alkaloids, phenolic compounds, and essential oils. The traditional uses are reviewed along with their pharmacological activities. Moreover, safety aspects are reported to provide a deep overview of the pharmacology potential of this genus. An updated search was carried out in databases such as Google Scholar, ScienceDirect, and PubMed to retrieve the information. Overall, this genus is a rich source of alkaloids of different types and also contains interesting phenolic compounds, such as anthocyanins, flavonols, and the characteristic indole derivatives nudicaulins. Among other pharmacological properties, numerous preclinical studies have been published about the analgesic, anticancer, antimicrobial, antioxidant, and antidiabetic activities of *Papaver* plants. Although it highlights the significant impact of this genus for the treatment of a variety of diseases and conditions, as a future prospect, characterization works accompanying preclinical studies are required along with clinical and toxicology studies to establish a correlation between the scientific and traditional knowledge.

## 1. Introduction

Many plants are naturally rich sources of phytochemicals with valuable biological properties, which could have significant impact for the treatment of a variety of diseases and conditions and as potential alternative options for synthetic drugs. This is also the case of the genus *Papaver* (family Papaveraceae), which is known for its medicinal properties attributed to their phytochemical composition ([1]; [180].

**Figure 1:**
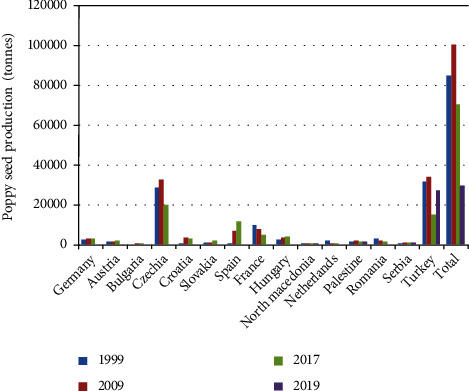
World production of poppy seeds in 1999, 2009, 2017, and 2019 according to FAOSTAT [8].

This genus belongs to the family Papaveraceae, which is a cosmopolitan family growing from tropical to alpine ecosystems [1], mainly in the northern hemisphere [2]. The flowers have no style, but on the top of the ovary, a stigmatic tissue is arranged radially on a sessile stigmatic disc. Their similar characteristics in their flower shapes, colors, and fruits complicate the identification based only on morphological characteristics [2], and different number of species is given in literature. For example, it consists of ~80–100 species, including annual, biennial, and perennial herbs [1, 3]. As the family, the genus *Papaver* is widely natural distributed, especially in regions with Mediterranean climate [1, 4]. In the case of *Papaver somniferum* L. (opium poppy), the most important species and due to its narcotic properties, it is highly produced in countries such as Afghanistan, Myanmar, Mexico, and Lao PDR (or Laos) [5], but illegally [6]. Alternatively, Turkey is one of the main legal manufacturers of the poppy plant [7], along with Czechia, Spain, etc.  Figure 1 shows the world production of poppy seeds in the last twenty years according to the data available from the Food and Agriculture Organization of the United Nations [8].

Other commonly cultivated species of the genus *Papaver* are *Papaver bracteatum* Lindl. (Iranian poppy), *Papaver rhoeas* L. (common poppy or corn poppy), *Papaver dubium* L., *Papaver pseudo-orientale* Medw., and *Papaver orientale* L. *P. bracteatum* that grow wild in high altitudes in north and northwest of Iran, in Russia and Caucasia regions [9]. *P. rhoeas* is an important competitive plant in winter cereals in southern Europe under Mediterranean climate [10] and thus called corn poppy. *P. dubium* is also called long-head poppy. *P. dubium* is widespread throughout Europe and America and is an important weed in western Iran [11]. *P. orientale* and *P. pseudo-orientale* are distributed into the Caucasus area [12].

Concerning the natural product field, *P. somniferum* was the first source of natural drugs with the obtainment of analgesic morphine drugs [13], including codeine, morphine, and a variety of semisynthetic derivatives, mainly derived from thebaine, such as oxycodone and buprenorphine [14]. These compounds belong to the opiate family that has analgesic properties mainly by binding to the mu-opioid receptor within the central nervous system (CNS) and the peripheral nervous system. It leads to an overall reduction of the nociceptive transmission [15]. However, the latex of the opium poppy is not only used for the treatment of severe pain, but it is also subjected to opioid abuse and drug trafficking due to the narcotic properties of these compounds. Therefore, their production is regulated internationally.

*P. bracteatum* has high content of thebaine as the main alkaloid, which has been utilized in the production of codeine [16]. Besides these compounds, the nonnarcotic papaverine is another economically important alkaloid with vasodilator properties [1, 17].

Apart from the alkaloids, poppy plant is a rich source of phenolic compounds, such as anthocyanins, flavonols, and the characteristic indole derivatives nudicaulins, and essential oil volatiles, which altogether are responsible for its pharmacological activities.

Moreover, *Papaver* seeds are esteemed in the food sector, e.g., to be used in bakery and desserts and to produce oil. For example, *P. somniferum* seeds are used in some Central Eastern European countries (European Food Safety Authority, [18]). In this context, poppy can be classified into three main categories depending on the use: industrial poppy intended for alkaloid extraction from the capsule of the plant; culinary poppy when it is grown to obtain seeds and oil; both industrial and culinary poppy [19]. *P. rhoeas* is also used as garniture in salad in some regions [20], and the seeds of *P. bracteatum* are used in foods in Central Anatolia [21].

Other different use is as ornamental plants like *P. orientale* and *P. pseudo-orientale* [2].

Moreover, besides the aforementioned applications for the main important alkaloids, the bioactive properties of the genus *Papaver* are wide. Antioxidant, antimicrobial, anticancer, anti-inflammation, neuroprotection, and maintenance of fertility are some of the important bioactivities of the *Papaver* genus plant extracts, as depicted in the following. In this context, this review describes the traditional uses of *Papaver* species, their phytochemical composition, and bioactive properties, describing preclinical and clinical studies. Moreover, safety aspects are well discussed with important case studies. The overall components and pharmacological activities of the review are well illustrated in Figure S1.

## 2. Databases, e-Resources, and Keyword Search

Various search engines for the survey of the literature were used to compile the scientific information included in the current review. In particular, Google Scholar, ScienceDirect, PubMed, and SpringerLink were used. Literature was retrieved from the books and international journals of highly cited publishers, including Elsevier, Springer, Frontiers, Wiley, and Taylor and Francis. Very few information was derived from national journals with no information of the ranking on the basis of citations. The keywords “*Papaver*”, “*Papaver somniferum*”, “opium poppy”, “opium”, “traditional uses”, “ethnopharmacology”, “bioactivities”, “biological activities”, “phytochemical profile”, and combinations of these words were used for deriving the particular information about the *Papaver* genus. Any article in the English language mentioning these keywords was included in the review article. The research articles, which were exclusively related to the agronomic traits of the *Papaver* genus, were excluded for compiling the information of the current review. Moreover, ChemBioDraw Ultra 12.0 (CambridgeSoft, Cambridge, MA, USA) was used to draw the chemical structures of the phytochemical compounds from the *Papaver* genus.

## 3. Ethnobotanical Uses of the *Papaver* Genus in Different Human Cultures

The traditional and medicinal properties of this genus have been documented since 3000 BC. The main analgesic compound “morphine” was isolated from *P. somniferum* L. by a German pharmacist “Sertüner” in 1905 [22]. The genus *Papaver* is not popular for traditional medicine due to narcotic and other side effects. Nevertheless, there are several uses such as anti-inflammatory, antidiabetic, analgesic, and remedy for cough and lung infection as described in traditional medicine and detailed in Table 1. This includes the use of the flowers, buds, seeds, fruits, and leaves or aerial parts of the most popular species of *Papaver* in different countries and cultures.

## 4. Phytoconstituents

The identification of the phytochemical composition of medicinal plants is highly important to provide a best known of the active compounds. It involves complex mixtures of natural compounds with different organic structures and varies depending on the plant source [48].

*Papaver* species contain alkaloids, phenolic compounds, and essential oil volatiles, among other components [49]. These classes found in different parts of the *Papaver* plants are discussed in the following subsections.

### 4.1. Alkaloids

**Figure 2:**
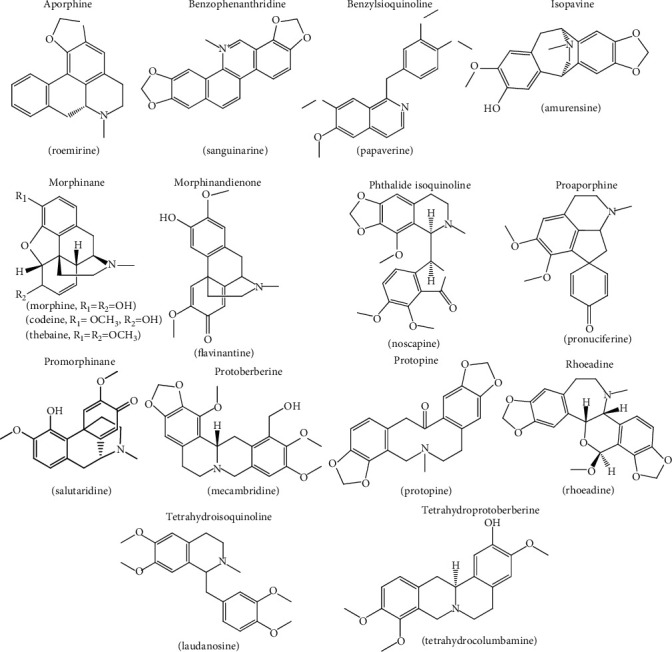
Selected alkaloids to exemplify the chemical structure of the different types found in the genus *Papaver*.

As other phytochemicals, the production of alkaloids in poppy plants is induced by environmental stress conditions, but the details about regulatory processes are not well known and subjected of ongoing studies [50]. Moreover, the alkaloid composition varies even within the same species [51]. This makes that the *Papaver* genus yields more than 170 alkaloids [52, 53]. As an example, Figure 2 summarizes the type of alkaloids found in the genus *Papaver* with their chemical structures.

In particular, *P. somniferum* presents interesting benzylisoquinoline alkaloids, such as papaverine, and the morphinanes morphine, codeine, and thebaine (Table 2, Figure 2), as mentioned in Section 1. Since *P. somniferum* has been extensively utilized illegally, its cultivation is strictly regulated by the International Narcotics Control Board [54, 55]. The latex of the opium poppy, which surrounds the seed capsule [56], may contain up to 80 alkaloids, but the latter compounds, morphine, codeine, and thebaine, along with narcotine and narceine are generally the main alkaloids [17, 18].

*P. bracteatum* is also a source of the alkaloid thebaine, the precursor of the opiate analgesics codeine, buprenorphine, oxymorphone, and oxycodone [14, 57, 58]. The plant capsule of this species shows high concentrations of morphine and oripavine (another morphinan) as compared to the stem tissues. It seems that the origin and even the latitude affect the thebaine, morphine, and oripavine [59]. This makes that it has intraspecies variation. For example, another study found that the major alkaloids in this species were salutaridine (promorphinan) and thebaine [53].

Concerning *P. rhoeas*, phytochemical composition has showed rhoeadine alkaloids as major compounds, including rhoeadine and rhoeagenine [60, 61]. Recent trends based on mass spectrometry (MS) analysis enabled the identification of a high number of phytochemicals from *Papaver* samples. For alkaloid profiling, electrospray ionization in the positive ionization modes generally leads to richer and complex chromatographic profiles with more intense signals for elucidation purposes [62]. Using this technique, for example, 55 alkaloids were characterized in the aerial parts of *P. rhoeas* and *P. somniferum*. This included benzophenanthridine, protoberberine, benzylisoquinoline, aporphine, and rhoeadine-type alkaloids (see examples, in Figure 2). The most characteristic feature was that rhoeadine alkaloids were observed only in *P. rhoeas* samples, and codeine and morphine were tentatively identified in *P. somniferum* [180] (Table 2).

In the latter work, different solvents were tested for extraction including ethyl ether with 10% ammonia, pure ethanol, and methanol, as well as aqueous-methanol 50% and 80%. Among them, ethanol can be applied to extract the aerial parts of *P. rhoeas* and *P. somniferum*, with advantages due to its high extraction efficiency [181] and as its low toxicity. Similarly, four *Papaver* species (*Papaver Lacerum* Popov, *Papaver syriacum* Boiss. & Blanche, *Papaver glaucum* Boiss. & Hausskn., and *P. rhoeas*) were collected from different sites in Turkey and the aerial parts were extracted using methanol. By using LC-tandem MS, two alkaloids, pronuciferine (proaporphine type) and roemerine (aporphine type), were determined in the selected species [63]. The latter compound was the major one in some *P. rhoeas* samples [64]. Recent studies showed that both alkaloids increase brain-derived neurotrophic factor (BDNF) protein expression in hippocampal SH-SY5Y cells demonstrating that besides the common poppy alkaloids, the former alkaloids could also be interesting [65]. Other compounds identified were salutaridine (promorphinan type), coulteropine (protopine type), and rhoeadine derivatives (epiglaucamine, glaudine, and rhoeagenine) [64]. Furthermore, using a combination of LC-MS and molecular networking, isoquinoline alkaloids in *Papaver nudicaule* L. and *P. rhoeas* aerial parts were clustered. 42 and 16 compounds were characterized, respectively, and a variation was observed depending on the color of the flowers [66].

*P. macrostomum*, which is widely distributed in Turkey, contains alkaloids such as protopine (protopine), benzylisoquinoline (macrostomine, dehydromacrostomine, sevanine), rhoeadine (rhoeadine, papaverrubine A-E), aporphine (isocorydine), isopavine (amurensine, amurensinine), protoberberine (cheilantifoline), proaporphine (mecambrine), and benzyl tetrahydroisoquinoline (laudanosine) types [67]. Moreover, the major alkaloids of *P. orientale* were oripavine (morphinan type) and mecambridine (protoberberine type) and of *P. pseudo-orientale* were also mecambridine and isothebaine (aporphine type) and orientalidine (protoberberine type). Main compounds of *Papaver duvium* L. are berberine and thalifendine, while roemerine is present in *P. lacerum*. The presence of isocorydine, stylopine (tetrahydroprotoberberine type) and tetrahydropseudocoptisine, roemerine, mecambrine, and allocryptopine depends on the subspecies [68]. The alkaloid composition of other less known *Papaver* species was described by Sariyar [53].

Rhoeadine is another group of alkaloids which is very common and widespread in the genus *Papaver* and contains at least 25 types. Particularly, alpinigenine, alpinine, and epialpinine were isolated from the *Papaver alpinum* L., whereas epiglaudine was isolated from the *P. glaucum*. Other rhoeadine-type alkaloids include glaucamine, glaudine, isorhoeadine, isorhoeagenine, isorhoeagenine-D-glucoside, *N*-methylporphyroxigenine, oreodine, oreogenine, papaverrubines A, B, C, D, E, F, G, H, rhoeadine, and rhoeagenine which are extracted from different species of *Papaver*. In general, all rhoeadines are characterized by a benzazepine system fused with six-membered acetal or hemiacetal moieties [69].

### 4.2. Phenolic Compounds

Phenolic compounds are natural antioxidants and other interesting phytochemicals found in *Papaver* plants. For example, petals of *P. rhoeas* flowers present flavonoids, which are responsible for their color, including white, yellow orange, white, and red colors. Particularly, the red flowers of this species contain anthocyanins [70]. This agreed with the results obtained by Soulimani et al. [71], who showed that a lyophilized ethanolic aqueous extract of *P. rhoeas* petals has anthocyanins, whereas no alkaloids were detected. Anthocyanins such as pelargonidin glycosides have been detected in red and orange petals of the plant [72].

**Figure 3:**
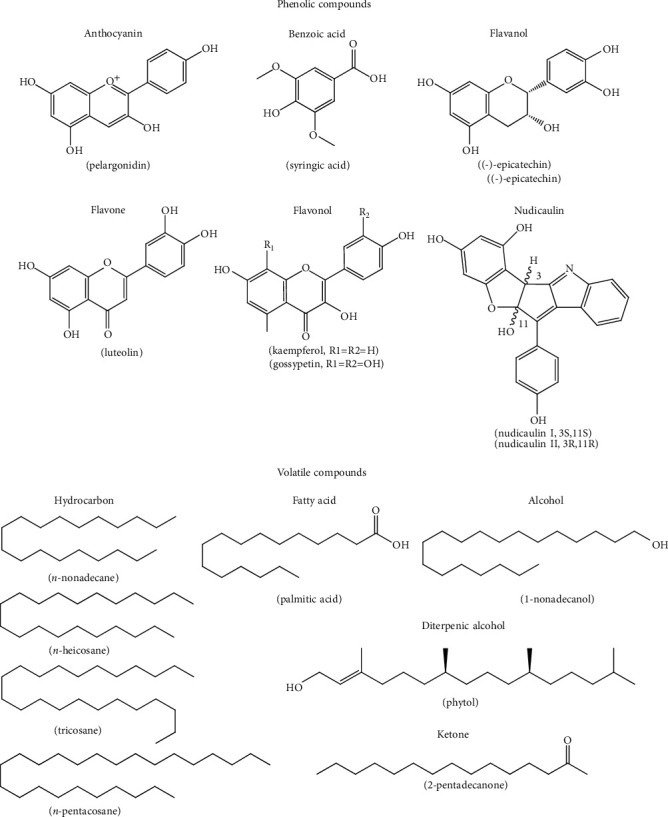
Phenolic compounds structures and main volatile compounds identified in *Papaver* plants.

In *P. nudicaule* cultivars, the flavonoid-derived indole alkaloids, nudicaulins, along with pelargonidin glycosides (anthocyanin), and kaempferol and gossypetin glycosides (flavonols) have been reported in the apical petals (Figure 3) [73, 74]. Other flavonoids such as gossypetin glycosides are present in the basal spot of all cultivars whereas carotenoids are present in yellow-colored stamens [73]. Another study found nudicaulins, gossypetin 7-*O*-glucoside (gossypitrin), and seven kaempferol glycosides in yellow petals of this plant [75]. Moreover, *Papaver alpinum* L. also accommodates some of these compounds [74].

Among the solvents, water, ethanol, and aqueous ethanol can be applied to extract high amounts of phenolics, but among them, the water extract showed the highest phenolic content. It was found that the aqueous extract of *P. somniferum* stalk contains high amount of phenolics, including flavonoids. The methanol and aqueous extracts presented considerable amounts of the flavanol *(−)*-epicatechin and the benzoic acid syringic acid [76]. Moreover, the aerial parts of *P. macrostomum* had the flavone luteolin (Figure 3) [67].

### 4.3. Essential Oils and Other Components

Dilek et al. [77] evaluated the essential oil composition of *P. somniferum* flowers after extraction by the hydrodistillation method. It mainly included *n*-nonadecane (9.0%), heneicosane (10.8%), *n*-pentacosane (7.9%), palmitic acid (7.3%), and 1-nonadecanol (16.3%) [77] (Figure 3). In another work, Krist et al. [78] identified the main volatile compounds in *P. somniferum* seed oil samples were 1-pentanol (3.3–4.9%), 1-hexanal (10.9–30.9%), 1-hexanol (5.3–33.7%), 2-pentylfuran (7.2–10.0%), and caproic acid (2.9–11.5%). It seems that the plant part could determine the composition of the volatile constituents, but little work has been done to investigate it.

The essential oil of the aerial parts of *P. rhoeas* that was gathered from the Elazig region in Turkey was obtained by hydrodistillation and analyzed using gas chromatography. Twenty-one constituents comprised the 98.6% of the total essential oil volatiles extracted from the plant. The major ones were phytol (52.8%), tricosane (7.8%), 2-pentadecanone (6%), and heneicosane (5.3%) (Figure 3); some of them are in common with *P. somniferum* [79]. Among them, the diterpene phytol is another interesting bioactive compound [80].

Moreover, the triglyceride composition of *P. somniferum* seed oil has been analyzed by matrix-assisted laser desorption/ionization-time-of-flight-MS and electrospray ionization ion trap-MS/MS. It enables the determination of the major triglyceride components, which were composed of linoleic, oleic, and palmitic acid, comprising approximately 70% of the oil [78]. The presence of high amount of unsaturated fatty acids makes the poppy seed oil suitable for its application in foods for maintaining the cardiovascular health.

### 4.4. Phytochemical Variation

The type of phytochemical and its content mainly depend on the part used and solvent applied for the extraction, as it was discussed in the previous sections. Also, intraspecific variation occurs [51, 59], for example, due to different locations [59], growth stage, and conditions [181]. This is extremely important for standardization or to choose those plants with strong enough potency to be applied to obtain functional ingredients.

For example, in a relevant study, empty poppy capsules (poppy straw) of 15 cultivars of *P. somniferum* were studied for the phytochemical profile. The seeds were raised in randomised block design with 3 replications during three consecutive years in 2007, 2008, and 2009. The extracts from the poppy straw were prepared using 5% acetic acid under sonication and then analyzed using liquid chromatography-MS. The overall results showed that the ratio of the alkaloids, morphine, codeine, narcotine, papaverine, and thebaine was highly variable in the selected 15 poppy cultivars, more than the difference found between the years [81].

## 5. Biological Activities of the *Papaver* Genus

*Papaver* forms part of the traditional system of medicines that plays an important role in providing health care to large section of the world population. Therefore, in this section, we discuss the updated snapshot of the bioactivities and therapeutic applications of *Papave*r genus, some of them related to its traditional use (Table 1).

### 5.1. Analgesic Activities

Few studies have already recognized that the treatment addressed to the immune system modulates the analgesic effect of the opiates isolated from poppy plant. It seems that during illness, the inhibition of morphine analgesia is due not only to the offsetting of analgesia by enhanced pain sensitivity but the action of endogenous antianalgesic mechanisms can be implied. The role of *N*-methyl-D-aspartate and central opioid receptors was established by Johnston and Westbrook [82], as well as the glial activation in the spinal cord. Other *Papaver* species have revealed some analgesia properties. Ibrar and group [83] reported the analgesic activity of the *Papaver pavoninum* C.A. Mey. extract. The study was completed on a mouse model, and the results demonstrated that plant extract significantly reduced pain in mice at all the three doses (50, 100, and 150 mg/kg body weight), as indicated by reduction in number of writhes as 36.91, 57.01, and 68.39%, respectively. The reduction in pain was dose dependent; hence, the 150 mg/kg dose proved to be most effective than the standard analgesic drug. Similarly, the ethanolic extract from the aerial parts of *Papaver libanoticum* Boiss., an endemic plant to Lebanon, exhibited a potent dose-dependent analgesic activity, which involved activation of opioid receptors in the central nervous system. This activity could be attributed due to the presence of alkaloids, different to morphine or its derivatives, and phenolic compounds [178].

Alternatively, besides to have mild opioid activity [178], Shams et al. [84] tested the effect of the administration of a hydroalcoholic extract from *P. rhoeas* to mice to evaluate the analgesic tolerance induced by morphine (1–10 mg/kg) using the tail-flick method. The results indicated that the extract of *P. rhoeas* showed no effects on analgesia at 25–100 mg/kg. However, treated animals with different doses of the extract (25–100 mg/kg) before the administration of morphine were effective to decrease the analgesic tolerance promoted by morphine.

### 5.2. Cytotoxicity Studies and Anticancer Activity

Several studies have shown that *Papaver* genus, including *P. somniferum*, *P. rhoeas*, *Papaver lacerum* Popov, and *P. nudicaule*, can provide anticancer compounds, but most studies were performed *in vitro* or in silico ([85, 86]; [179]; [87–89]), as shown in Table 3. Their efficacy depends again on the part and solvent used [88]. Moreover, among the studied compounds, alkaloids have shown anticancer properties [86, 87]. Nonetheless, the most active alkaloids were berberine and macranthine; importantly, they demonstrated low toxicity against the Vero cell line, a noncancerous model. *P. somniferum*-based nanoparticles (PbO and Fe_2_O_3_) have shown cytotoxicity in HepG2 cell lines in order to treat hepatic carcinoma [87]. PbO-based nanoparticles demonstrated higher cytotoxicity (~79% inhibition) owing to more penetration due to its smaller size as compared to Fe_2_O_3_ nanoparticles (61% inhibition).

In another work, the chemical extracts from the petals of *P. rhoeas* have recently been tested for potential in the prevention of skin cancer. Sublethal UVB-mediated lesions at both DNA and RNA levels in human keratinocytes were observed, and thus, derived sunscreen based on the extracts of *Papaver* petals could be promising [90]. As commented before, petals can have phenolic compounds, other potential active compounds.

The lethality to brine shrimp can be applied as prescreen to existing cytotoxicity and antitumor assays [91]. In this context, other studies have tested *Papaver* extracts in brine shrimp eggs [91, 92]. It was established that the most active extract was obtained from *P. pavoninum* whole plant extracted with ethanol (lethal concentration 50% or LC_50_ = 2.54 *μ*g/mL) compared to *P. rhoeas* seed extracts obtained with dichloromethane (LC_50_ = 24 *μ*g/mL) and methanol (LC_50_ = 26 *μ*g/mL) [83, 92]. Since the latter LC_50_ values were lower than 30 *μ*g/mL, these extracts displayed significant cytotoxicity, according to Khalighi-Sigaroodi et al. [91], who tested extracts from other 23 plant species of the Leguminosae family.

Concerning *in vivo* studies, cytotoxicity has been mainly focused on concrete alkaloids and also the mechanisms of action studied in cancerous cell lines. Besides the aforementioned studies, the nonnarcotic alkaloids noscapine and papaverine have been found as potent anticancer agents against different human cancers such as breast, liver, bone, prostate, colorectal, and fibrosarcoma by inhibiting the cell proliferation, inducing apoptotic cell death, and causing cell cycle arrest [93].

Noscapine has been found to suppress the cell proliferation, migration, and invasion as well as also induce apoptosis. The supplementation of noscapine at the rate of 320 *μ*M concentration to human skin cancer cell line (A-431) induced 80% cell death and induced the structural change in human serum albumin protein [94, 95]. Noscapine also presents strong anticancer potential against human epithelial ovarian and prostate cancers via inducing apoptosis in a receptor-dependent but radical oxygen species- (ROS-) independent manner [96]. Noscapine has anticancer activity against two LNCaP and PC-3 human prostate cancer cell lines, but it was combined with paclitaxel. This combination produced significantly lowering the mRNA expression of B-cell CLL/lymphoma (Bcl-2) and increasing the mRNA expression of Bcl-2-associated X protein (Bax), and Bax/Bcl-2 ratio, among other effects [97]. In this regard, the apoptosis of cancerous cells is regulated by the members of the Bcl-2 family (Bax, Bcl-2). Bcl-2 factors inhibit the apoptosis whereas Bax factors promote it; hence, the ratio of both the factors decides the fate of cancerous cells. Noscapine also improved its therapeutic anticancer potential in colon cancer SW480 cells through inducing apoptotic cell death by blocking the liver-intestine cadherin (CDH17) gene. It also shows a significant effect on the levels of proteins related to apoptosis (Cyt-c, Bax, Bcl-2, and Bcl-xL) [98]. In human SW480 colon cancer cells, noscapine markedly decreased the colony-forming ratio and cell viability, up-regulated the expression levels of cleaved-poly (ADP-ribose) polymerase and cleaved-caspase-3, inhibited cell proliferation, and promoted cell apoptosis [99]. Alternatively, another study proved that noscapine has been found effectively to inhibit proliferation and invasion of MG63 cell line by suppressing the phosphorylation of epidermal growth factor receptor (EGFR) gene and its downstream pathway [100].

There are also numerous studies on the anticancer effects of papaverine in cells. For example, papaverine exhibited anticancer activity on human glioblastoma (GBM) temozolomide (TMZ; as a first-line anticancer medicine)-sensitive U87MG and TMZ-resistant T98G cells via preventing tumor cell growth, suppressed cell migration, and significantly inhibited the cell proliferation. It was also reported that papaverine has a dose-dependent cytotoxic effect on human prostate cancer cells (PC-3) through inducing early and late apoptosis along with inducing sub-G1 cell cycle arrest, lowering the expression levels of Bcl-2 proteins, increasing the Bax protein levels, reducing the NF-*κ*B levels, and downregulating the PI3K and phospho-Akt expression [101, 102]. This observation is in line with Antonarakis et al. [103] who also reported other mechanisms such as an enhancement in the expression levels of Bax protein, the release of cytochrome C into the cytoplasm, reduction in the expression levels of X-linked inhibitor of apoptosis protein, and induction of apoptosis. Papaverine was also found effective against hepatic carcinoma by inhibiting the telomerase through downregulation of telomerase reverse transcriptase in humans in HepG-2 cells [104]. Likewise, noscapine and papaverine have an anticancer effect on human MCF-7 and MDA-MB-231 cell lines via enhancing apoptosis, causing cell cycle at G2/M phase, and arresting cell cycle at G_0_/G_1_ phase [105].

Moreover, papaverine in combination with low-frequency ultrasound improved the blood-brain barrier, which is involved in the maintenance of brain homeostasis and compromised in brain tumors [106, 107]. This combination was able to reduce the expression levels of zonula occluden-1, occludin, and claudin-5, enhancing the permeability of blood-tumor barrier. This can be a strategy for selective crossing this barrier by chemotherapeutic drugs [107]. Another *in vivo* study showed that papaverine also markedly delayed the tumor growth in a U87MG xenograft mouse model [108, 109].

Besides the latter compounds, sanguinarine is another promising anticancer compound effective against a variety of multidrug-resistant cancers and combined with chemotherapeutic agents to synergistically enhance their sensitivity [110]. Also, berberine has shown anticancer potential in cells ([87]; [179], among others, as Table 3 shows.

### 5.3. Antimicrobial Activity and Antiviral Activities

The antimicrobial activity of several extracts from *Papaver* plants is shown in Table 4. Among these studies, *P. somniferum* seed extracts, containing alkaloids and phenolic compounds, among other components, have shown the highest antimicrobial activity for the methanol extract against *Staphylococcus aureus* and *Aspergillus* species [111], whereas the aqueous and ethanolic extracts against root rot fungi at 5% [112]. In another work, AMA of *P. somniferum* in nanosystem was evaluated when it was used for the green synthesis of nanoparticles based on lead oxide (PbO) and iron oxide (Fe_2_O_3_). Both the nanoparticles resulted in effective antimicrobial activity against all the pathogenic microbial strains (*Bacillus subtilis*, *Staphylococcus epidermidis*, *Klebsiella pneumoniae*, *Pseudomonas aeruginosa*, *Fusarium solani*, *Aspergillus flavus*, *Aspergillus fumigates*, and *Aspergillus niger*) in a dose-dependent manner (4 to 10 mg/mL concentration) [102]. However, *Papaver*-based fabrication of PbO nanoparticles resulted in higher antibacterial property due to its small size than Fe_2_O_3_-based nanoparticles.

In a comparison study performed by Ünsal and coworkers [113], the antimicrobial extracts obtained with various solvents from the aerial parts of *P. argemone*, *P. dubium*, *P. rhoeas*, and *Papaver clavatum* Boiss. & Hausskn. ex Boiss. were recently investigated. Among the solvent tested, *P. dubium* extracted by petroleum ether and diethyl ether showed a higher effectiveness against *S. aureus*, with a minimum inhibitory concentration (MIC) of 9.76 and 19.52 *μ*g/mL, respectively, compared to chloroform, ethanol, and acetone. Even, lower values have been reported for the tertiary alkaloids obtained from the aerial parts of *P. rhoeas* when it was tested against six bacterial species (*S. aureus, S. epidermidis*, *Escherichia coli*, *K. pneumoniae*, *P. aeruginosa*, and *Proteus mirabilis*), and three *Candida* strains (*C. albicans*, *C. parapsilosis*, and *C. tropicalis*) were studied using a microbroth dilution method. In this study, the plant samples were collected from 11 different sites, obtaining the best antimicrobial activity against *S. aureus* and *C. albicans* with an MIC value of 1.22 and 2.42 *μ*g/mL in the site with the higher content of roemerine alkaloid [64]. Additionally, Table 4 displays the antimicrobial activity of other *Papaver* species. Among them, the results of *Papaver pseudocanescens* M. Pop extracts as an antiviral agent seem promising [114].

In a similar way, the antiviral activities of active compounds of *P. rhoeas* pollen against influenza H1N1, H3N2, and H5N1 viruses have been evidenced. Total, six flavonoids, including kaempferol derivatives and luteolin, and one alkaloid, chelianthifoline, were isolated and revealed neuraminidase inhibitory activities, reducing the ability of the virus to spread. The concentration required for 50% inhibition (IC_50_) ranged from 10.7 to 100.5 *μ*M for H1N1, 25.6 to 143.2 *μ*M for H3N2, and 12.6 to 151.1 *μ*M for H5N1. Among all tested compounds, luteolin was found to be the most active [115]. The antimicrobial activity of nudicaulin derivatives (synthesized *in vitro* and *in vivo* in *P. nudicaule*) has also been evaluated, but only one derivative (17-methyl-5,7,11,3′,4′-penta-*O*-methylnudicaulin) was slightly active [86].

### 5.4. Antioxidant Activity

The *in vitro* antioxidant activity of *P. somniferum* has been reported by using different methods, including the 2,2-diphenyl-1-picrylhydrazyl (DPPH), 2′-azinobis-(3-ethylbenzothiazoline-6-sulfonate) (ABTS), and chelating assays [119] (Table 5). Zhang and coworkers from China described the antioxidant activity of the powdered poppy capsule extractive by using DPPH assay and its relationship with quantitative fingerprinting. Morphine and codeine were among the components that have a positive influence in this bioactivity [120]. This agreed with the results obtained by other authors [121]. Moreover, a recent study evaluating different parts of the plant suggests that the flower extract (rich in anthocyanins) and leaves showed the highest antioxidant activity depending on the antioxidant assay. Although it correlated with the phenolic content, the alkaloid extract showed the highest antioxidant values, with inhibitory concentration (IC_50_) of 7.4 and 8.1 *μ*g/mL in the DPPH and ABTS radical scavenging activity assays [122]. In another context, the antioxidant activity of PbO and Fe_2_O_3_ nanoparticles synthesized using *P. somniferum* was evaluated. Using free radical scavenging assay (FRS), total reducing power assay (TRP), and total antioxidant capacity assay (TAC), it was observed that both the nanoparticles of *P. somniferum* exhibited concentration-dependent activity. PbO nanoparticles revealed the significant antioxidant activity in terms of FRS (54%), TRP (16.8 mg ascorbic acid equivalents/mg), and TAC (106.1 mg ascorbic acid equivalents/mg) while Fe_2_O_3_ nanoparticles showed 52% FRS activity, 16.8 mg ascorbic acid equivalents/mg TRP, and 131.1 ascorbic acid equivalents/mg TAC, respectively [102].

The antioxidant activity *in vivo* of *P. somniferum* has been also evaluated through the seed oil administered to rats, observing limited oxidative damage [123]. Concerning other species, the antioxidant activity of *P. rhoeas* has also been reported by three methods: DPPH, ABTS, and ferric reducing antioxidant power (FRAP) assays. The results clearly indicated that leaf extract demonstrated significant antioxidant activity with a half-maximal effective concentration (EC_50_) 28.72 mg/100 g dry weight in DPPH, 185.29 mM Fe^2+^/100 g DW in FRAP, and 12.07 mM Trolox equivalents (TE)/100 g dry weight in ABTS [124]. The water extract of this plant has also been evaluated for antioxidant potential using DPPH and superoxide anions assays. The IC_50_ value of *P. rhoeas* was 4.81 mg/mL for DPPH assay, and it was the highest antioxidant activity in the anti-ROS assay [125]. Moreover, the hydrophilic and lipophilic antioxidant activity of *P. rhoeas* was studied by using the TEAC assay. The leaves of wild *P. rhoeas* displayed the highest total antioxidant activity (1326 *μ*mol TE/100 g fresh weight) among other assessed plant species, which correlated with their total phenolic and flavonoid content [126].

The antioxidant action of *Papaver* plants depends on the genotype as shown by Krošlák and coworkers. Their results suggested that there were differences in the antioxidant activity of *P. somniferum* seeds by using DPPH, ABTS, FRAP, and reducing power (RP) assays. The genotype major displayed the best antioxidant activity in all the assays, namely, DPPH (126.29), ABTS (31.05), FRAP (31.61), and RP (146.56) mg of TE. Alternatively, the genotype MS-423 showed the high inhibition against trypsin, thrombin, and collagenase enzymes [127]. Other important factor is the solvent used for extraction. The results by Selen Isbilir & Sagiroglu [20] indicated that water extract (WE) of *P. rhoeas* was the most effective compared to ethanol (EE), and acetone extracts (AE); total antioxidant activity of all the extracts was recorded to be 96.01% (WE), 94.98% (EE), and 89.07% (AE), respectively. The TRP of extracts was as follows: WE > EE > AE [20]. Other studies on antioxidant potential of *Papaver* genus are presented in Table 5, showing the selected solvent for extraction. Although it is difficult to compare all the solvents due to the use of different assays and units, it seems that the methanolic extract was higher in antioxidant activity compared to other solvents for the aerial parts of *P. bracteatum*.

From the aforementioned studies and Tables 4 and 5, it can be summarized that the bioactivity of the alkaloids derived from the *Papaver* genus depends on the extraction conditions, as the phytochemical composition. Extraction condition may include the type of the solvent, extraction time, temperature, and other input factors. In addition, *Papaver* alkaloids or phytochemical extracts demonstrated more effective bioactivities in the nanoforms. Smaller nanoparticles penetrate more easily into the bacterial membrane and dissociate into respective ions causing oxidative stress, membrane leakage, and killing bacterial cells with more perfection. The use of well-established alkaloids for the treatment of various ailments in the human body may be utilized as nanoformulation to enhance the efficacy of the drug. There is further need to develop the field of nanotechnology with respect to *Papaver-*based drug formulations. In addition, testing other isolated compounds is required to assess the antimicrobial activity and antioxidant potential and their contribution in order to select most active plant extracts.

### 5.5. Antidiabetic Activity

It is well known that *α*-amylase and *α*-glucosidase are key enzymes for the catabolism of complex carbohydrates into glucose and thus target to explore antidiabetic drugs. In this sense, the *α*-glucosidase inhibitory activity of *P. somniferum* seeds (aqueous and ethanol extracts) was also demonstrated. It was found that both the extracts showed less than 5% inhibitory activity [132]. Also, the *α*-amylase enzyme inhibition activity using *P. somniferum* pod-based PbO and Fe_2_O_3_ nanoparticles showed insignificant inhibition as 3% and 25%, respectively [102].

Apart from this, large number of researchers documented the antidiabetic potential of this genus in literature through traditional medicine knowledge [133–136]. Most of these studies referred to *P. rhoeas* and particularly to the seeds. For example, boiled seeds capsule of *P. rhoeas* were used by the communities with 22.85% frequency based on the information collected by 35 healers in Iran [37]. Alternatively, the antidiabetic effects of opium were low in experimental diabetic animals at an oral dose of 10 mg/kg body weight for 90 days, as reported by Ahmed and group. Although opium increased serum insulin and decreased serum glucose, the effect was not significant; this was due to metabolic disorders in diabetic animals. In addition, it is suggested that opium consumption in diabetic patient is not useful [137]. Similarly, Sadeghian et al. [138] reported the effects of available opium substance on glucose and lipid metabolism in streptozotocin-induced-diabetic rats by testing opium contained in the juice of the seed capsule of the *P. somnniferum*. The test rats were treated with normal opium (20 mg), starting on the fifth day after induction of diabetes for 30 days. The results demonstrated that glycaemia levels in the rats treated with opium (544.8 mg/dl) were similar to the levels determined in the control rats (524.6 mg/dL). In addition, the level of other parameters was similar: serum, total cholesterol, high-density lipoprotein, and triglyceride. Indeed, more studies are needed to clarify the role of *Papaver*, specifically, *P. rhoeas*, in the antidiabetic action and the active chemical components.

### 5.6. Properties in Fertility

The role of *P. rhoeas* extract (dried petals macerated with 50% ethanol) on fertility has also been investigated in mouse oocytes [139]. The cumulus-oocyte complexes were cultured in a maturation medium supplemented with different concentrations (low: 10-25 *μ*g/mL; high: 50-200 *μ*g/mL) of *P. rhoeas* extract. Low concentrations of extract showed moderate effects; however, higher concentration (100 *μ*g/mL) significantly improved the rate of oocyte maturation and embryo development in mouse oocyte maturation medium [139]. In another study, similar findings were obtained while working on sheep oocytes [140]. The results demonstrated that plant extract displayed dose-dependent activity in a maturation medium. The concentration of 50 *μ*g/mL was effective and improved the sheep oocyte maturation rate when the extract was supplemented in a maturation medium [140]. Flavonoids, including anthocyanins, have been associated with these effects, which can protect intracellular glutathione levels in oocytes [141].

### 5.7. Neurological/Mental Effects

Supplementation of *P. rhoeas* hydroalcoholic extract reduced depression and increased the neurotransmitters involved in depression, including dopamine, serotonin, and norepinephrine [142]. Depression is also linked with stress and increases glucocorticoid secretion into the blood. Nonetheless, the administration of a hydroalcoholic extract of *P. rhoeas* (15-60 mg/kg in male mice) enhanced the secretion of glucocorticoids, but it could reduce the side effects of stress [143]. In other work, *P. rhoeas* distillate decreased anorexia and improved learning ability, but it again increased the corticosterone levels [144]. These positive neurological/mental effects agreed with recent results that suggest that *P. rhoeas* hydroalcoholic extract has a reducing effect on depression in mice after short-term administration. In this sense, the antidepressant effect of *P. rhoeas* may not be due to the inhibition of the hypothalamic-pituitary-adrenal stress system, while it could be caused, at least in part, by the inhibition of glutamate or through antiopioid and anticholinergic effects [145].

Furthermore, sedative effects of *P. rhoeas* aqueous and alcoholic extracts have also been observed, being more marked when 10% ethanol was used as solvent for extraction [71].

### 5.8. Other Bioactivities

The antiulcerogenic activity of *P. rhoeas* root extract was assessed by using the ethanol-induced ulcerogenesis model in rats. The plant extract (670 mg/kg) exhibited statistically significant (95.6%) gastroprotective effects. In addition, histopathological studies confirmed the positive results of the extract *in vivo* [146]. The anti-inflammatory activity of extracts from *P. nudicaule* aerial parts and its mode of action in RAW264.7 macrophage cells have been also tested. Interestingly, in this work, the aerial parts were selected according to different colors (white, orange, yellow, scarlet, and pink) and under two different growth stages (after 60 and 90 days). All of the extracts of *P. nudicaule* displayed significant effects in reducing lipopolysaccharide- (LPS-) induced nitric oxide; the white flower extract-90 showed the best results. This extract also decreased the LPS-induced nitric oxide synthase 2 and cyclooxygenase 2. It inhibited the LPS-induced activation of nuclear factor-*κ*B and signal transducer and the activator of transcription 3 signalling pathway [147]. As commented before, the phenolic composition depends on the color and the white ones have petals rich in kaempferol glycosides, but with a lack of pelargonidin glycosides and nudicaulins [73]. Moreover, *P. rhoeas* extracts prevented pain and inflammation due to their potential activity on opioid, glutamate, and nitric oxide systems, as well as elevated the plasma corticosterone concentration [148]. Furthermore, *P. somniferum* seeds have inhibitory activities against trypsin, thrombin, and collagenase, suggesting more vast pharmacological possibilities [127].

## 6. Safety and Adverse Effects

An undesired harmful effect resulting from a medication or other intervention such as surgery is known as an adverse effect. It may be termed a “side effect,” when considered to be secondary to the therapeutic effect [149]. In contrast, dangerous, unintended reactions of medicines that occur at doses normally used for treatment are called adverse drug reactions (ADRs), even can lead to death in many countries [149]. In the case of morphine alkaloids, the pharmacologic properties of these compounds differ widely and their medicinal applications have some safety and adverse effects [150].

Various wanted and unwanted effects of opium consumption are discussed in the encyclopedia Canon of Medicine by Avicenna (980-1037 AD). Avicenna has mentioned on the mechanism of opioid-related respiratory depression, due to respiratory muscle spasm for respiratory failure. Similarly, it is mentioned in Canon of Medicine that opium can cause abnormal and difficult breathing, which can lead to death. A respiratory suppression side effect was observed with patients suffering from fever associated with tuberculosis due to the use of the topical opioid application on the chest. Constipation and painful bowel obstruction were other adverse effects of opium-based [177]. Avicenna has also mentioned poisoning, sluggishness, sedation, and abdominal contractions. Opium has highly addictive qualities and is reported to cause memory and reasoning dysfunction [177].

Remarkably, modern studies have confirmed the adverse effect of morphine alkaloids described by Avicenna [151–153]. Some of them are related to the binding to *μ*- and *κ*-opioid receptors, the accumulation of neuroexcitatory opioid metabolites, etc. One of the side effects of opioid-based pain relievers, including morphine and its derivatives, is severe constipation [154]. Kohberg et al. [155] and Rocker et al. [156] also reported constipation as the most frequent adverse effect. Moreover, other effects of morphine are on the CNS mediated by its high affinity to the *μ*-opioid receptor, such as nausea, vomiting, sedation, euphoria, miosis, respiratory depression, drowsiness, and obstipation [157]. Additional adverse effects are endocrinopathies and sleep disorders. Furthermore, long-term use of opioid can lead opioid tolerance (increased dose needed for analgesia) and hyperalgesia (paradoxical increase in pain with opioid administration) that involves *μ*-opioid receptor signalling pathways [158–160]. Wound healing can also be delayed by chronic morphine intake by inhibiting immune cell recruitment followed by wounding [161].

As codeine is a precursor of morphine, they share some pharmacological features with also direct activity at the opioid receptors, but the former has much lower potency. The most frequent side effects of codeine are constipation and nausea, and addition potential. Nevertheless, in some paediatric patients, the genotype predisposing to ultrarapid metabolism of codeine into morphine by the isoenzyme CYP2D6 can occur [157]. Codeine and morphine can be distributed into breast milk with complications for breastfed infants of mothers receiving codeine [162], even a case of severe neonatal toxicity in a breastfed infant has been reported [163]. In the case of noscapine, it is used as a centrally acting antitussive compound and no toxicological properties have been characterized, but it can present headache and dizziness [157].

Poppy seeds from *P. somniferum* are commercially available in some countries and widely used as ingredients for various kinds of food, especially in Eastern Europe [164]. Poppy seeds for food uses are generally obtained from cultivars bred to accumulate lower amounts of opium alkaloids [56] and normally contain low levels (2-251 *μ*g/g of morphine and 0.4-57 *μ*g/g of codeine) [165]. The opioid concentrations come primarily from the alkaloid residue retained on the seeds [166]. Therefore, although the consumption of poppy seeds in foods is really in small amounts, EFSA set a general safe level of 10 *μ*g per kilogram of body weight based on the morphine content of poppy seeds [157]. In this sense, only a rare case of death has been published consuming between 64 and 587 times the volume of poppy seeds (around 900 g). This extremely high ingest led to death due to complications of a bowel obstruction, but it did not cause lethal opiate toxicity [165].

Alternatively, extracts or infusions concentrated in opium alkaloids from poppy seeds can have adverse effects [56, 165], but there are few reports on this topic [166]. In any case, some authors have attempted the reduction of the content of opium alkaloids in the seeds using different treatments. For example, while the levels of opium alkaloids were not affected by baking or steam application, a high reduction of these compounds can be obtained by water washing or extended thermal treatment [56].

In another context, immunoglobulin E-mediated sensitization to poppy seeds is rare, but if it occurs, the clinical symptoms can be severe, e.g., due to cross-sensitizations events [167, 168].

Concerning other *Papaver* species, some case studies in humans suggest that unconscious ingestion of *P. rhoeas* can cause acute liver toxicity [169] and intoxication with different effects (nausea, restlessness, dyspnoea, contractions unconsciousness, numbness, etc.) [170]. Alternatively, an *in vivo* study performed by Soulimani and coworkers [71] suggests that extracts from *P. rhoeas* petals (without the presence of alkaloids) showed a lethal dose (LD_50_) of 4000 mg/kg and thus very low toxicity. However, sedative effects were observed. A study *in vitro* showed that *P. rhoeas* leaf extract also showed promising antimutagen/anticlastogen activity [171] and thus suggesting low toxicity. Therefore, although *Papaver* extracts can have some beneficial effects, toxicity studies are further required to establish dosage and side effects. Nonetheless, the culinary use of some parts and *Papaver* plants indicates that the safety issues are controversial or the dosage is a prerequisite. This includes *P. somniferum* seeds, with the aforementioned exceptions [56, 165]; the shoots of *P. rhoeas*, the aerial parts of this species, and *Papaver strictum* Boiss. & Balansa are added to salads, minestra, etc. [32, 35, 172]. Additionally, in Turkey, poppy flowers are used as food colorant and for enhancing the flavour of herbal teas [64].

## 7. Clinical Trials

Besides the aforementioned case reports studies, there are a very limited number of clinical studies reporting the health beneficial effects of *Papaver* plants, as far as we know. One of them tested the iodized poppy-seed oil as vehicle of the drug epirubicin against hepatocellular carcinoma [173, 174], but the anticancer effects of poppy have not been evidenced in humans. In the ClinicalTrials.gov database, there are two studies based on the administration of California poppy (*Eschscholzia californica Cham.*) (NCT03364101) but only one refers to the *Papaver* genus. In this work, ground poppy seeds were baked into a bran muffin and administered to evaluate the effect on postprandial blood glucose response, vascular, appetite, and sensory parameters (NCT01579656), but the results have still not been posted. Furthermore, a recent study on *P. rhoeas* combined with other herbs in syrup has improved sexual experience of men following consumption of this mixture with no drug-related serious adverse events. Therefore, the authors suggest that this aphrodisiac syrup can be applied alternatively to other chemical sexual drive enhancers with complicated side effects [175].

## 8. Conclusions and Future Perspectives

Besides the pharmacological interest of *P. somniferum*, the traditional use of different *Papaver* plants is widely established in different cultures and countries. This fact makes this genus attractive as a source of pharmacoactive extracts and compounds (alkaloids, phenolic compounds, and essential oil). These compounds are responsible for the multifaceted biological activities of the *Papaver* genus including anticancer, antioxidant, antimicrobial, and analgesic. The finding from different studies also demonstrated that these useful compounds are present throughout the plant including agro-residue generated from the *Papaver* plants. Nonetheless, pharmacological studies on extracts from these plants should be reinforced with characterization studies to know the active molecules, or if synergism exists that makes more interesting the use of the whole extracts. For that, bioassay-guided fractionation or even chemometrics with MS-based methodologies and HPLC with MS/MS can be applied to identify the overall profile of the *Papaver* plant extracts. This is especially important since the phytochemical composition and content as well as the bioactivity depend on several factors, including the genotype, the growth stage, and even the color of the flower. The phytochemical profile also depends on the method of extraction, input factors used for the extraction, and also on the style of preparation of the sample for analysis. Moreover, little is known about the bioactivity of the essential oil from these plants, even though some authors suggest the presence of phytol. This compound is valuable as a fragrance and exhibits a broad range of bioactivities [80].

Moreover, applications of this genus in nanotechnology seem promising, for example, to synthesize nanoparticles for different pharmacological purposes but further work is required, including more toxicity studies. In this sense, the use of plant extracts is increasing in green synthesis and the type of compounds present on these extracts can modulate the nanoparticle shape (Vijayaraghavan et al., [176]) and probably its functionality. Finally, although some preclinical results are promising, more clinical studies are needed to provide scientific evidence of the traditional use of *Papaver* plants before consumption and to avoid intoxication events. Overall, these studies along with a better known of the active molecules through comprehensive characterization and bio-guided fractionation studies should be undertaken in future research.

## Figures and Tables

**Table 1:** Traditional medical usage of *Papaver* species.

*Papaver species*	Country	Internal/external usage	References
*P. argemone* L.	Iran	Flower (headache, coughs)	Naghibi et al. [23]
*P. bracteatum* Lindl.	Iran	Flowers, leaves, seeds (hypnotic and sedative, respiratory tract infections, sore throat, food digestion, eyelid inflammation, spasm, rheumatism pains)	Farouji and Khodayari [24]
*P. dubium* L.	Turkey	Flower (colds, cough)	Altundaga and Ozturkb [25]; Çakılcıoğlu et al. [26]
*P. lacerum* Popov	Turkey	Buds (goiter)	Altundaga and Ozturkb [25]
*P. lateritium* K. Koch	Turkey	Flower (sedative, antitussive, bronchial, hypnotic)	Akbulut and Bayramoglu [27]
*P. macrostomum* Boiss. & A.Huet	Turkey	Flower (cough)	Altundaga and Ozturkb [25]
*P. orientale* L.	Turkey	Seed (laxative); leaf (asthma)	Altundaga & Ozturkb [25]
*P. rhoeas* L.	Turkey	Herb (sedative); fruit, seed (gastrointestinal diseases) External usage: aerial part (red spots on body); fruit (burns); seed (dermal diseases, wound)	Altundaga and Ozturkb [25]; Çakılcıoğlu et al. [26]; Polat and Satıl [28]; Ugulu [29]; Yipel et al. [30]
	Italy	Fruit, young shoot (sedative, hypnotic); young aerial part (depurative); leaf, flower (mental-nervous, hypnotic, mild sedative for child, cough)	González-Tejero et al. [31]; Mattalia et al. [32]; Naghibi et al. [23]; Pieroni, [33]; Pieroni and Quave [34]; Scherrer et al. [35]; Vitalin et al. [36];
	Algeria	Aerial part (respiratory diseases)	Gonzalez-Tejero et al. [31]
	Cyprus	Aerial part (nervous/mental conditions, digestive)	Gonzalez-Tejero et al. [31]
	Spain	Aerial part (respiratory diseases)	Gonzalez-Tejero et al. [31]
	Iran	Seed, capsule (antidiabetic); flower (addiction, sedative, hypnotic)	Bahmani et al. [37]; Nadaf et al. [38]
*P. somniferum* L.	Turkey	Fruit, seed (gastrointestinal, nervous and respiratory diseases)	Yipel et al. [30]
	Italy	Fruit, seed (tranquiliser, toothaches)	Pieroni and Quave [34]
	India	Seed (demulcent, spasmolytic, muscle catch, tonic); fruit (cough, diarrhea) External usage: leaf (swelling)	Jadnav [39]; Dar et al. [40]; Goyal [41]; Tayade and Patil [42]
	Pakistan	Herb (narcotic, stimulant, to increase performance, cough suppressant); fruit, leaf (analgesic, narcotics); seed (narcotic, analgesic, sedative, increases excitement and physical vigor)	Adnan et al., [43]; Alamgeer et al. [44]; Irfan et al. [45]; Ullah et al. [46]
	Korea	External usage: fruit, latex, stem (furuncle)	Kim and Song [47]

**Table 2:** Alkaloids characterized in *Papaver rhoeas* L. and *Papaver somniferum* L. by mass spectrometry in different locations. Adapted from [181].

Name	Formula	Mass (Da)	MS/MS fragments (*m*/*z*)	Alkaloid type	PR (R)	PR (SK)	PS
DL-Demethylcoclaurine^∗^	C_16_H_17_NO_3_	271.12	107.05, 255.10, 161.06, 143.05	Benzylsioquinoline	+	+	+
Coclaurine^∗^	C_17_H_19_NO_3_	285.14	107.05, 269.12, 175.07, 237.09	Benzylsioquinoline	+	+	+
Tetrahydropapaverine^∗^	C_20_H_25_NO_4_	343.18	192.10, 189.09, 151.08, 327.16	Benzylsioquinoline	-	-	+
Reticuline^∗^	C_19_H_23_NO_4_	329.16	192.10, 137.06, 143.05, 175.07	Benzylsioquinoline	+	+	+
Corytuberine^∗^	C_19_H_21_NO_4_	327.15	265.09, 237.09, 297.11, 205.06	Aporphine	+	+	+
Tetrahydrocolumbamine^∗^	C_20_H_23_NO_4_	341.16	178.09, 163.06, 176.07	Protoberberine (THPB)	-	-	+
Scoulerine^∗^	C_19_H_21_NO_4_	327.15	237.09, 207.04, 211.08, 239.07	Protoberberine (THPB)	-	-	+
L-Tetrahydropalmatine^∗^	C_21_H_25_NO_4_	355.18	192.10, 165.09, 176.07	Protoberberine (THPB)	+	-	+
Tetrahydroberberine (canadine)^∗^	C_20_H_21_NO_4_	339.15	176.07, 149.06, 174.05	Protoberberine (THPB)	+	-	+
Berberine^∗^	C_20_H_18_NO_4_	336.12	320.09, 292.10, 321.10, 306.08, 278.08	Protoberberine	+	+	+
Stylopine^∗^	C_19_H_17_NO_4_	323.12	176.07, 149.06	Protoberberine (THPB)	+	+	+
Dihydrosanguinarine^∗^	C_20_H_15_NO_4_	333.10	318.08, 319.08, 304.10, 276.10	Benzophenanthridine	+	+	+
Sanguinarine	C_20_H_14_NO_4_	332.09	317.07, 274.09, 304.10	Benzophenanthridine	+	-	-
Protopine^∗^	C_20_H_19_NO_5_	353.13	188.07, 189.08, 149.06	Protopine	+	+	+
Allocryptopine^∗^	C_21_H_23_NO_5_	369.16	188.07, 189.08, 290.09	Protopine	+	-	-
Morphine	C_17_H_19_NO_3_	285.14	201.09, 229.08, 185.06, 211.07	Morphinan	-	-	+
Mecambrine	C_18_H_17_NO_3_	295.12	202.09, 171.07, 280.10	Proaporphine	-	-	+
Codeine	C_18_H_21_NO_3_	299.15	215.11, 243.10, 225.09, 199.07	Morphinan	-	-	+
(S)-*N-*Methylcoclaurine	C_18_H_21_NO_3_	299.15	269.12, 107.05, 271.13	Benzylisoquinoline	+	+	+
Armepavine	C_19_H_23_NO_3_	313.17	107.05, 58.07, 269.12, 271.13, 298.11	Benzylisoquinoline	+	+	+
(S)-3′-Hydroxy-*N*-methylcoclaurine	C_18_H_21_NO_4_	315.15	192.10, 123.04, 285.11, 300.12	Benzylisoquinoline	+	+	+
(S)-Cheilanthifoline	C_19_H_19_NO_4_	325.13	178.09, 190.09, 163.06	Protoberberine	+	+	+
Papaverine	C_20_H_21_NO_4_	339.15	202.09, 324.12, 296.13, 171.07	Benzylisoquinoline	-	-	+
Cryptopine	C_21_H_23_NO_5_	369.16	352.12, 205.11, 165.09, 190.09	Protopine	+	+	+
Noscapine	C_22_H_23_NO_7_	413.15	220.10, 353.10, 365.10, 179.07	Phthalide isoquinoline	+	-	+
Codeinone	C_18_H_19_NO_3_	297.14	283.12, 282.11, 254.12, 266.12	Morphinan	+	-	-
Morphine *N*-oxide	C_17_H_19_NO_4_	301.13	284.13, 241.09	Morphinan	-	-	+
Flavinantine	C_19_H_21_NO_4_	327.15	178.09, 163.06	Morphinandienone	-	-	+
8,14-Dihydroflavinantine (or salutaridinol)	C_19_H_23_NO_4_	329.16	285.11, 123.04, 58.07, 143.05	Morphinan	+	+	+
(S)-*cis*-*N*-Methylstylopine	C_20_H_20_NO_4_	338.14	191.09, 190.09, 149.06	Protoberberine	+	+	-
Isocorydine	C_20_H_23_NO_4_	341.16	297.11, 265.09, 237.09	Aporphine	+	+	+
Pseudoprotopine	C_20_H_19_NO_5_	353.13	188.07, 189.08, 149.06	Protopine	+	+	-
Amurensinine *N*-oxide A (or amurensinine *N*-oxide B)	C_20_H_21_NO_5_	355.14	190.06, 191.09, 277.09, 151.08	Isopavine	+	+	+
Rheagenine (or isorheagenine)	C_20_H_19_NO_6_	369.12	352.12, 340.13, 324.12	Rhoeadine	+	+	-
Rhoeadine (or isorhoeadine)	C_21_H_21_NO_6_	383.14	321.08, 303.06, 291.07, 366.13	Rhoeadine	+	+	-
Glaucamine (or isoglaucamine)	C_21_H_23_NO_6_	385.15	368.15, 338.10	Rhoeadine	+	+	-
Coptisine	C_19_H_14_NO_4_	320.09	292.10, 277.07, 290.08, 318.08, 262.09	Protoberberine	+	+	+

PR: *Papaver rhoeas*; PS: *Papaver somniferum*; THPB: tetrahydroprotoberberine; RS: Russia; SK: South Korea; ^∗^univocally identified through comparison with standards.

**Table 3:** Cytotoxicity of the *Papaver* genus.

Species/extract name	Design/model	Key effects	Countries	References
*P. somniferum* L. Lead and iron oxide nanoparticles	*In vitro* study HepG2 cell lines	(i) PbO NPs showed higher cytotoxicity (20.9%) as compared to Fe_2_O_3_ NPs (38.5%) (ii) The cytotoxicity of whole plant extract (57.6%) was lower than both NPs	Pakistan	[102]
*P. Lacerum* Popov	*In vitro* study HeLa cell line *In silico* study	(i) Two compounds, namely, tyrosol-1-*O*-*β*-xylopyranosyl-(1→6)-*O*-*β*-glucopyranoside) (I) and 5-*O*-(6-*O*-*α*-rhamnopyronosyl-*β*-glucopyronosyl) mevalonic acid (II), were isolated from this species (ii) Both compounds exhibited modest cytotoxic effect, IC_50_ = 66.4 *μ*M and 54 *μ*M, respectively (iii) *In silico* study showed that protein-tyrosine kinase Syk and aldo-keto reductase family-1 were the targets, respectively	Turkey	[85]
*P. nudicaule* L. (nudicaulin and derivatives) Methanol–water	*In vitro* study HeLa, HUVEC and K-562 cell lines	(i) Synthetic nudicaulin derivatives 6–11 showed high antiproliferative activity against HUVEC and K-562 cells (ii) Derivative compounds showed significant cytotoxic activity against HeLa cells	Germany	[86]
*P. rhoeas* L. Ethanol extract	*In vitro* study HCT116, MCF7, HaCaT, and NCM460 cell lines	(i) The compounds stylopine, canadine, sinactine, berberine, and epiberberine and the raw extract showed a dose-dependent inhibitory effect. The highest activity was found for compound berberine against all cell lines (HCT116: IC_50_ = 90 *μ*M; MCF7: IC_50_ = 15 *μ*M; HaCaT: IC_50_ = 50 *μ*M; NCM460: IC_50_ ≥ 200 *μ*M)	Lebanon	[179]
*Papaver* alkaloids (amurine, armepavine, berberine, isocorydine, isothebaine, macranthine, mecambrine, mecambridine, narkotine, orientalidine, oripavine, salutaridine, and thebaine)	*In vitro* study HeLa, and Vero cell lines	(i) Berberine and macranthine were the most active alkaloids in all 13 compounds (ii) Dose-dependent studies were applied and revealed IC_50_ values of 12.08 *μ*g/mL (HeLa) and 71.14 *μ*g/mL (Vero) for berberine, and 24.16 *μ*g/mL (HeLa) and IC_50_ of >300 *μ*g/mL (Vero) for macranthine	Turkey	[87]
*P. somniferum* L. Hexane, methanol, and ethyl acetate	*In vitro* study HT29, HeLa, C6 cells, and Vero cell lines	(i) The inhibitory effects of the leaf, root, stem, and capsule extracts were shown on cancer cell lines (ii) The extracts were able to destroy cellular membrane in tumor cell lines at high concentrations (iii) Stem ethyl acetate extract exhibited strong anticancer activity on all cell lines, with IC_50_ values ranged from 119 to 391 *μ*g/mL), depending on the plant part and solvent	Turkey	[88]
*P. rhoeas* L. Methanol extract	*In vitro* study TK6 cell lines	(i) The highest inhibition of cell growth was observed at the concentrations of 5 mg/mL and 25 mg/mL after the treatment with plant extract	Slovakia	[89]
*P. pavoninum* Fisch & Mey. Ethanol extract	*In vitro* study Brine shrimp eggs	(i) The plant extract was found to produce outstanding dose-dependent cytotoxicity in terms of LC_50_ = 2.54 *μ*g/mL (ii) The dose concentration of 100 and 1000 *μ*g/mL produced high cytotoxicity as 83.3% and 96.7% lethality, respectively	Pakistan	[83]
*P. rhoeas* L. *n*-Hexane, dichloromethane, and methanol	*In vitro* study Brine shrimp eggs	(i) Dichloromethane and methanol extracts showed significant toxicity activity in brine shrimp lethality assay in terms of LC_50_ 24 and 26 *μ*g/mL, respectively	United Kingdom	[92]

IC_50_: 50% inhibitory concentration; LC_50_: lethal concentration 50%; NPs: nanoparticles.

**Table 4:** Antimicrobial activity of *Papaver* plants.

Species/extract name	Microbial strains	Key results	Assay	Country	References
*P. somniferum* L. Hexane, methanol, ethanol, and ethyl acetate extract	*Bacillus cereus* MTCC 430	0.14 mm ZOI	Disc-diffusion	India	[111]
	*Staphylococcus aureus* MTCC 3160	2.00 mm ZOI			
	*Escherichia coli* MTCC 40	0.10 mm ZOI			
	*Salmonella typhi* MTCC 3224	0.13 mm ZOI			
	*Aspergillus niger* MTCC 281	3.00 mm ZOI			
	*Aspergillus oryzae* MTCC 624	3.00 mm ZOI			
	*Aspergillus flavus* MTCC 227	1.50 mm ZOI			
	*Penicillium chrysogenum* MTCC 6795	2.00 mm ZOI			
*P. pseudocanescens* M. Pop Ethanol extract	Poliovirus type 1 (LSc-2ab)	21.4-49.7 *μ*M IC_50_	—	Bulgaria	[114]
	Human rhinovirus type 14 (HRV-14)	65-199 *μ*M IC_50_			
*P. rhoeas* L. Methanol, ethanol, water, and alcoholic-water extract	*Bacillus subtilis* ATCC 6633	—	Disc-diffusion	Serbia	[70]
	*Staphylococcus aureus* ATCC 6538	12-18 mm ZOI			
	Escherichia coli ATCC 8739	17-24 mm ZOI			
	Pseudomonas aeruginosa ATCC 9027	11-20 mm ZOI			
	Salmonella abony NCTC 6017	—			
	Aspergillus niger ATCC 16404	13-26 mm ZOI			
	*Candida albicans* ATCC 10231	—			
*P. somniferum* L. bee pollen Ethanol extract	*Penicillium citrininum*	4-5 mm ZOI	Disc-diffusion	Slovak	[116]
	*Penicillium crustosum*	4-9 mm ZOI			
	*Penicillium expansum*	1-4 mm ZOI			
	*Penicillium brevicompactum*	1-3 mm ZOI			
	*Penicillium chrysogenium*	—			
	Enterobacteriaceae	6-7 mm ZOI			
	*Staphylococcus* sp.	5-6 mm ZOI			
*P. argemone* L. subsp. davisii Petroleum ether, diethyl ether, chloroform, acetone, and ethanol extract	*Staphylococcus aureus* ATCC 65538	39-625 (*μ*g/mL) MIC	Microbroth dilutions	Turkey	[113]
	*Staphylococcus epidermidis* ATCC 12228	312-1250 (*μ*g/mL) MIC			
	Escherichia coli ATCC 25922	1250 (*μ*g/mL) MIC			
	*Klebsiella pneumonia* ATCC 4352	1250 (*μ*g/mL) MIC			
	*Pseudomonas aeruginosa* ATCC 27853	625-1250 (*μ*g/mL) MIC			
	*Proteus mirabilis* ATCC 14153	1250 (*μ*g/mL) MIC			
	*Candida albicans* ATCC 10231	312-625 (*μ*g/mL) MIC			
*P. clavatum* Boiss. & Hausskn. ex Boiss Petroleum ether, diethyl ether, chloroform, acetone and ethanol extract	*Staphylococcus aureus* ATCC 65538	78-156 (*μ*g/mL) MIC	Microbroth dilutions	Turkey	[113]
	*Staphylococcus epidermidis* ATCC 12228	312-625 (*μ*g/mL) MIC			
	*Escherichia coli* ATCC 25922	312-625 (*μ*g/mL) MIC			
	*Klebsiella pneumonia* ATCC 4352	—			
	*Pseudomonas aeruginosa* ATCC 27853	—			
	*Proteus mirabilis* ATCC 14153	625 (*μ*g/mL) MIC			
	*Candida albicans* ATCC 10231	625 (*μ*g/mL) MIC			
*P. dubium* subsp. lecoqii var. lecoqii Petroleum ether, diethyl ether, chloroform, acetone, and ethanol extract	*Staphylococcus aureus* ATCC 65538	9-1250 (*μ*g/mL) MIC	Microbroth dilutions	Turkey	[113]
	*Staphylococcus epidermidis* ATCC 12228	312-625 (*μ*g/mL) MIC			
	*Esc*h*erichia coli* ATCC 25922	1250 (*μ*g/mL) MIC			
	*Klebsiella pneumonia* ATCC 4352	625-1250 (*μ*g/mL) MIC			
	*Pseudomonas aeruginosa* ATCC 27853	625-1250 (*μ*g/mL) MIC			
	*Proteus mirabilis* ATCC 14153	625-1250 (*μ*g/mL) MIC			
	*Candida albicans* ATCC 10231	625 (*μ*g/mL) MIC			
*P. rhoeas* L. Petroleum ether, diethyl ether, chloroform, acetone and ethanol extract	Staphylococcus aureus ATCC 65538	39-156 (*μ*g/mL) MIC	Microbroth dilutions	Turkey	[113]
	*Staphylococcus epidermidis* ATCC 12228	156-625 (*μ*g/mL) MIC			
	*Escherichia coli* ATCC 25922	625-1250 (*μ*g/mL) MIC			
	*Klebsiella pneumonia* ATCC 4352	—			
	*Pseudomonas aeruginosa* ATCC 27853	—			
	*Proteus mirabilis* ATCC 14153	625 (*μ*g/mL) MIC			
	*Candida albicans* ATCC 10231	625 (*μ*g/mL) MIC			
*P. somniferum* L. Aqueous and ethanol extract	*Fusarium solani*	13-20 mm ZOI	Paper disc	Pakistan	[112]
	*Rhizoctonia solani*	15-24 mm ZOI			
	*Macrophomina phaseolina*	15-22 mm ZOI			
	*Fusarium solani*	18-25 mm ZOI	Well method		
	*Rhizoctonia solani*	15-24 mm ZOI			
	*Macrophomina phaseolina*	21-29 mm ZOI			
*P. macrostomum* Boiss. & A.Huet Petroleum ether, diethyl ether, chloroform, acetone, and ethanol extract	*Staphylococcus aureus* ATCC 6538	1-14 mm ZOI	Disc-diffusion	Turkey	[67]
	*Staphylococcus epidermidis* ATCC 12228	5-32 mm ZOI			
	*Escherichia coli* ATCC 11229	1-7 mm ZOI			
	*Pseudomonas aeruginosa* ATCC 1539	2-9 mm ZOI			
	*Proteus mirabilis* ATCC 14153	1-16 mm ZOI			
	*Klebsiella pneumoniae* ATCC 4352	1-6 mm ZOI			
	*Candida albicans* ATCC 10231	3-6 mm ZOI			
	*Candida glabrata* ATCC 90030	5 mm ZOI			
	*Candida guilliermondii* KUEN 998	6 mm ZOI			
	*Candida tropicalis* KUEN 1021	2-4 mm ZOI			
	*Candida pseudotropicalis* KUEN 1012	5 mm ZOI			
	*Candida krusei* ATCC 6258	1-4 mm ZOI			
*P. decaisnei* Hochst. & Steud. ex Elkan Methanol extract	*Bacillus subtilis* ATCC 6633	Non-significant	Microbroth dilutions	Iran	[117]
	*Candida albicans* ATCC 10231	-			
	*Escherichia coli* ATCC 10536	Non-significant			
	*Klebsiella pneumoniae* ATCC 10031	-			
	*Morganella morganii* PTCC 1078	-			
	*Pseudomonas aeruginosa* ATCC 4027	Non-significant			
	*Salmonella typhi* PTCC 1185	-			
	*Staphylococcus aureus* ATCC 29737	-			
*P. rhoeas* L. Ethyl alcohol extract	*Bacillus subtilis* ATCC 6633	+	Microbroth dilutions	India	[118]
	*Escherichia coli* ATCC 10536	+			
	*Saccharomyces cerevisiae* ATCC 9763	+			

IC_50_: inhibitory concentration at 50%; MIC: minimum inhibitory concentration; ZOI: zone of inhibition; -: not active; +: active.

**Table 5:** Antioxidant activity of *Papaver* plants.

Species and type of extract	Assay	Key results	Countries	References
*P. rhoeas* L. Methanolic extract	DPPH	IC_50_ = 1.4 mg/mL	Slovakia	[89]
*P. somniferum* L. Ethanolic extract	Total reducing power	3592.56 mg/mL	Serbia	[116]
*P. rhoeas* L. Ethanolic extract	DPPH	81.47–89.71%	Serbia	[70]
*P. rhoeas* L. Methanolic extract	CUPRAC ABTS/persulfate FRAP	0.13 mmol TR/g 0.15 mmol TR/g 0.07 mmol TR/g	Turkey	[128]
*P. somniferum* L. Methanolic extract	Linoleic acid peroxidation	49.75 IC_50_ (*μ*g/mL)	Iran	[129]
*P. bracteatum* Lindl Methanolic extract	Linoleic acid peroxidation	IC_50_ = 3.51 *μ*g/mL	Iran	[130]
*P. rhoeas* L. Aqueous methanol extract	DPPH H_2_O_2_ Fe^2+^	EC_50_ = 63.01 (*μ*g/mL) 10.57–52.70% 86.85%	Turkey	[131]

ABTS: 2,2′-azino-bis(3-ethylbenzothiazoline-6-sulfonic acid; CUPRAC: cupric reducing antioxidant capacity; DPPH: 2,2-diphenyl-1-picrylhydrazyl; EC_50_: half-maximal effective concentration; EC_50_: half-maximal effective concentration; FRAP: ferric-reducing antioxidant power; TR: Trolox.

## Abbreviations

EFSA: European Food Safety Authority
IC_50_: Inhibitory concentration at 50%
EC_50_: Half-maximal effective concentration
LC_50_: Lethal concentration 50%
MIC: Minimum inhibitory concentration
MS: Mass spectrometry
ZOI: Zone of inhibition.
